# Caution

**Published:** 1855-09

**Authors:** 


*Caution.*—Persons desirous of attending the Lectures of the "College of Physicians and Surgeons of the Iowa University" will if they desire to avail themselves of the benefits of Scholarships, apply to the Members of the Legislature of this State, and to other officers, including the Judiciary. This was an arrangement entered into for the benefit of those of our citizens who desired to complete their Medical education, but who might be unable to meet the expenses attending. Recently we accidentally met with what purported to be a Scholarship, and as persons may be deceived, without a particular examination, into the supposition that they emanated from this department; we take this occasion to warn all who desire to attend the State Institution, against the reception of any Certificate unless it bears the impression of the College buildings, the names of the Trustees and Faculty, and the name of J. C. Hughes, M. D., Dean of the Faculty. None else are the genuine issues of this Faculty.

